# Phthalate Exposures in the Neonatal Intensive Care Unit

**DOI:** 10.3390/toxics9050090

**Published:** 2021-04-21

**Authors:** Randall Jenkins, Devlynne Ondusko, Luke Montrose, Ryan Forbush, David Rozansky

**Affiliations:** 1 Department of Pediatrics, Oregon Health & Science University, Portland, OR 97239, USA; Ondusko@ohsu.edu (D.O.); Rozansky@ohsu.edu (D.R.); 2 Department of Community and Environmental Health, Boise State University, Boise, ID 83725, USA; lukemontrose@boisestate.edu; 3 Department of Respiratory Care, Boise State University, Boise, ID 83725, USA; ryanforbush@boisestate.edu

**Keywords:** hypertension, prematurity, phthalates, di-2-ethylhexyl phthalate (DEHP), toxicity

## Abstract

Background: Di-2-ethylhexyl phthalate (DEHP), a phthalate compound found in medical devices, may cause toxic effects in premature infants. In this study, the objective is to quantify DEHP exposures from various intravenous and respiratory therapy devices, and to use these values to predict typical exposure for an infant in a neonatal unit. Methods: Common IV products used on infants are directed through various types of IV tubing (IVT) and analyzed for DEHP content. DEHP exposure for infants receiving respiratory therapy was determined indirectly through analysis of urine DEHP metabolites. By deriving these values for DEHP we calculated the daily exposure to DEHP from common IV fluids (IVF) and respiratory devices during hospitalization in a neonatal unit. Results: IVF labeled DEHP-positive showed very high concentrations of DEHP, but when passed through IVT, substantial amounts were adsorbed. DEHP was undetectable with all DEHP-negative IVF tests, except when passed through DEHP-positive IVT. The DEHP leached from most respiratory devices was relatively modest, except that detected from bubble CPAP. In 14 very low birthweight infants, the mean DEHP exposure was 182,369 mcg/kg over 81.2 days of the initial hospitalization. Ninety-eight percent of the exposure was from respiratory devices, with bubble CPAP accounting for 95% of the total DEHP exposure in these infants. Conclusions: The DEHP exposure in our neonatal unit can be reduced markedly by avoiding or modifying bubble CPAP equipment and avoiding IV tubing containing DEHP.

## 1. Introduction

Phthalates are a group of more than 25 synthetic organic compounds derived from phthalic acid [1,2]. Phthalates are used as industrial solvents and plasticizers and are incorporated in a multitude of industrial and home products including personal care products and medical devices [1,2,3,4]. Humans are exposed to phthalates from food ingestion, skin absorption, and from inhalation [3]. A comprehensive review of biomonitoring of human phthalate exposure worldwide has been recently published [1]. Phthalate exposures in children is higher than that in adolescents and adults [5].

Toxicity concerns have been raised including cancer risk, followed by reproductive and developmental risks, as well as other endocrine abnormalities [1,6,7]. Premature infants are disproportionately exposed to phthalates because of their intensive need for medical devices, immature metabolism, and low body weights [7,8]. Reports of phthalate-related neurodevelopmental abnormalities, endocrine disruption (including genital anomalies), and hepatic injury have also appeared [9,10,11,12,13,14,15]. Recently our group reported an association between phthalate exposure and hypertension in premature infants [16]. Our results suggest phthalates may cause hypertension through activation of the mineralocorticoid receptor via inhibition of 11 *β*-HSD2, the enzyme which converts cortisol into the less potent cortisone [16]. Inhibition of 11 *β*-HSD2 by monoester phthalate metabolites has also been shown in human microsomes [17].

Di-2-ethylhexyl phthalate (DEHP) is the only phthalate approved by the FDA for use in medical devices in the United States. DEHP is added to polyvinyl chloride (PVC) devices to make them flexible, soft and durable [1,2,3]. DEHP does not covalently bind to PVC and can easily leach from PVC devices into a contact medium, subsequently reaching patients via vasculature, inhalation, or mucous membranes [1,3,6,8]. Outside the United States, particularly in Europe, alternative plastic polymers and alternative phthalates are used in medical devices [18].

The principal metabolite of DEHP is mono (2-ethylhexyl phthalate (MEHP), which is rapidly oxidized into two monoester metabolites, mono-(2-ethyl-5-hydroxyhexyl) phthalate (MEOHP) and mono-(2-ethyl-5-oxyhexyl phthalate (MEHHP) [8]. Two other metabolites with a longer half-life are 2-(carboxymethyl) hexylphthalate (2cx-MMHP), and 2-ethyl-5-carboxy phenylphthalate (5cx-MEPP) have been identified. In an adult, 67% of an ingested DEHP dose was excreted in the urine in 24 h, making urine metabolites good biomarkers for DEHP exposure. Similar excretion data is not available for premature infants [8].

In the newborn intensive care unit (NICU), DEHP has been identified and measured for a variety of individual items used in the NICU over the past 20 years [19,20,21,22,23,24]. Based on these measurements, during a typical NICU stay, Mallow estimated a 2 kg premature infant would receive a DEHP exposure of 16.3 mg/kg/day, exceeding safe levels by 3–5 orders of magnitude [7]. A recent evaluation showed that almost all DEHP-labeled products in the NICU are either intravenous (IV) devices (bags and tubing) or respiratory therapy tubing (including endotracheal tubes) [16]. Given the ongoing risk to premature infants from DEHP exposures and the availability of some devices without DEHP, we sought to quantify current DEHP exposures to an especially at-risk population, very low birthweight (VLBW) infants in the NICU. The main objective was to produce estimates of specific DEHP exposures and to show the range and magnitude of these exposure to the VLBW infants in the NICU.

## 2. Materials and Methods

### 2.1. Overview

The study design includes direct in vitro measurement of DEHP emanating from various IV sources as well as indirect estimation of DEHP leaching from respiratory devices into premature infants. The indirect estimates were based on in vivo excretion of DEHP metabolites using Koch’s method [25]. Using both our direct and indirect DEHP exposure values, we calculated the cumulative DEHP exposure during the initial hospitalization of fourteen consecutive VLBW infants who were admitted into a single NICU based on a chart review of all identified IV and respiratory DEHP exposures.

### 2.2. In Vitro DEHP Measurements

Sham IV setups were used to directly measure DEHP that leaches from PVC IVF containers and associated PVC tubing sets currently found in NICUs. Three to seven tests were done for each test item. Fluid items tested included three brands of commercial IVF, pediatric hyperalimentation fluid (HA), and three types of lipid emulsions. Commercial IVF tests were performed with normal saline (0.9% sodium chloride) as this was a common commercial IVF given to premature infants. Of the three brands of IVF tested, one brand was labeled to contain DEHP (DEHP-positive), a second brand was labeled DEHP-negative), and a third lacked any label referring to DEHP (DEHP-unlabeled). Three types of lipid emulsions were tested: fish-oil-based, soybean-based, and a product with a mixture of fish oil, soybean oil, olive oil, and medium-chain triglycerides (from here forward referred to as a mixed lipid emulsion). Samples were collected in glass containers and assayed for DEHP by gas chromatography/mass spectrometry by ALS Environmental Corp., Kelso, WA, USA.

Tubing sets were of two types, referred herein as microbore and standard tubing. Microbore (small-bore) tubing was used to deliver fluid from syringes to patients. Standard tubing was used to administer IVF from fluid bags to infants using a linear peristaltic pump. Two brands of microbore tubing, both labeled DEHP-negative were tested. Two standard tubing sets were tested and were identical in physical dimensions and appearance, but one was DEHP-positive and the other was DEHP-negative. Testing was done on fluid directly collected from the fluid’s container, as well as that collected after passage through tubing sets. When testing commercial IVF or HA, the fluid flow rate was set at 20 mL/hour for 24 h, simulating a clinical scenario for IV fluid delivery which might be given to a 3.0 kg infant at a rate of 160 mL/kg/day. The lipid flow rate was dependent on the lipid emulsion used: for mixed and soybean-based 20% lipid emulsions, the rate was set to reflect a dose of 3 g/kg/day (1.8 mL/h for 24 h); whereas for fish oil-based 10% lipid emulsions, the rate was set to reflect a dose of 1 g/kg/day (2.5 mL/h for 12 h). The combinations tested are shown in Table 1.

### 2.3. In Vivo DEHP Determinations

The method by Koch predicts that 44.2% of a DEHP exposure will be excreted over 24 h in the form of three key metabolites, MEHP, MEOHP, MEHHP [25]. Excretion of these DEHP metabolites can be extrapolated to daily intake (DI) for DEHP using the following equation from David [26], as modified by Koch [8]:

DI (g/kg_body weight_/day) = (UE (mol/g × CE (g/kg_body weight_/day)/FUE × MW_DEHP_

where UE is the urinary excretion of the monoester metabolite (in mol/g creatinine), CE is the creatinine excretion rate normalized by body weight, FUE is the urinary excretion ratio (0.442 for the sum of the three main metabolites (MEHP, MEOHP, MEHHP).

Urine creatinine excretion was calculated by the method used by Modi and Hutton, which predicts creatinine excretion for premature infants based on age and weight [27]. The equation is:

Creatinine excretion (µmol kg^−1^ day^−1^) = 55.2 + 0.13 postconceptional age (days).

Urine samples were obtained from VLBW premature infants receiving one of the following respiratory therapy devices: low-flow nasal cannula, high-flow nasal cannula, bubble CPAP, and a conventional ventilator. In order to achieve steady state excretion, samples were not obtained until the therapy had been stable for at least 3 days. Each devise comprised respiratory tubing, identical humidification chambers, and a DEHP-free (labeled) cannula or endotracheal tube. Five samples per device were obtained from three or more unrelated patients if possible. The number of days on each device prior to sampling was recorded. We excluded samples where there were other IV DEHP exposures aside from the respiratory device. Three samples were not excluded because the daily calculated DEHP exposure from IV fluid was less than 1 mcg.

We excluded sampling from patients who were diagnosed with acute kidney injury or chronic kidney disease. Urine was collected using cotton balls placed in the diaper. Samples were subdivided and frozen at −80 C. DEHP urine metabolites were measured in a commercial lab (SGS Lab in Sidney, B.C.) using high performance liquid chromatography with tandem mass spectrometry.

### 2.4. Statistical Analysis

Direct and indirect DEHP exposures were described using median exposure with interquartile range due to non-normal distributions of data. The presence or absence of DEHP in IVT was compared for significant difference using the Wilcoxson ranked sum test. This test was also used to compare estimated DEHP exposure from respiratory devices as compared with the room air (baseline) exposure.

### 2.5. Estimated DEHP Exposure in an NICU

Charts of VLBW infants in one NICU were analyzed for DEHP exposures. A diagnosis of secondary hypertension, acute kidney injury, or chronic renal disease resulted in the infant’s exclusion from analysis. The first 14 infants, which were not otherwise excluded, were selected for data analysis. All IV and respiratory therapy exposures were tabulated each day from birth until either 40 weeks postmenstrual age or discharge, whichever came first. Based on our above-derived in vitro testing of IV exposures, the daily volume of each type of IV exposure was expressed as micrograms of DEHP exposure. Based on our above-derived in vivo estimation of DEHP exposure, the daily respiratory exposures were also expressed as micrograms of DEHP exposure. Median values from our in vitro and in vivo studies were used to calculate the daily DEHP exposure for each individual exposure. Exposure in mcg/kg was calculated for each day and for the cumulative hospitalization.

## 3. Results

### 3.1. In Vitro DEHP Measurements

Measurements of DEHP which leached from various brands of commercial IVF containers and tubing sets are shown in Table 1. Raw data appears in the Appendix A. Large variation was found among brands of commercial IVF tested. We found measurable amounts of DEHP in all DEHP-positive IVF, and from all IVF after passage through DEHP-positive IVT. All tests from DEHP-negative IVF—either directly obtained from the bag, or when passed through DEHP-negative tubing—revealed no DEHP. When DEHP-negative IVF was passed through DEHP-positive IVT, DEHP was documented in the fluid after such passage. DEHP concentrations measured in DEHP-unlabeled IVF were intermediate to the concentrations found in DEHP-negative or -positive IVF.

The concentration of DEHP measured directly from the DEHP-positive IVF bag was far more than that measured after transit of the IVF through either the DEHP-negative or -positive IVT. HA fluid as well as fish-oil based lipid emulsions deliver similar amounts of DEHP as commercial IVF when administered through DEHP-negative tubing sets. However, when delivered through DEHP-positive IV tubing, DEHP measurements for HA fluid and lipid emulsions were approximately 100-fold and 1000-fold that measured in commercial IVF respectively.

### 3.2. In Vivo DEHP Determinations

Urine samples were obtained from 12 infants, but only 21 samples from eight of these infants were analyzed. The remaining samples were excluded due to multiple IV and respiratory DEHP exposures creating unclear attribution. Samples testing CPAP and baseline (no exposures) patients were from three and four unrelated infants respectively. Samples from high-flow nasal cannula patients were from two siblings only. Samples testing ventilator patient exposures were from one patient. The mean number of days the patients were on the respiratory device before urine sampling was 13 (see Appendix B). Urine DEHP metabolites were measurable in all samples. DEHP exposure values derived from these measurements are shown in Table 2. For those samples obtained in infants with no identifiable DEHP exposures, we labelled the derived values as “room-air baseline” exposure. Derived DEHP exposure for low-flow and high-flow nasal cannula were similar to the baseline DEHP exposure. DEHP exposure from ventilators were two- to threefold higher than the baseline DEHP exposure but not statistically different. Bubble CPAP DEHP exposure was more than 100-fold higher than ventilator or baseline exposures, *p* < 0.05.

### 3.3. Estimated DEHP Exposure in an NICU

Demographics and DEHP Exposures estimated for the 14 VLBW infants are shown in Table 3 and Table 4. The mean cumulative exposure was 230,207 mcg. The mean daily exposure was 2306 mcg/kg/day. For the group, 98% of the exposures were from respiratory DEHP and 97% of the respiratory DEHP exposures were from bubble CPAP therapy.

## 4. Discussion

This investigation used in vitro sham IV systems to measure DEHP leaching from intravenous products, and in vivo measurement of urine DEHP metabolites to estimate DEHP exposure from respiratory devices. Respiratory exposures were highly variable, but variations were based on the type of therapy used and the DEHP content of the endotracheal tube. IV DEHP exposures were generally smaller than respiratory DEHP exposures, and were heavily dependent on the DEHP content of the tubing, varying by orders of magnitude. Although the results do not directly predict levels of harm, they provide information to prioritize ways to reduce DEHP exposures in the NICU. Since bubble CPAP accounted for 95% of the mean DEHP exposures, finding a way to alter this exposure would make a large reduction in DEHP exposures. Alterations could include different tubing, different humidification water, or a different choice of respiratory therapy device. Intravenous exposures (especially from HA and lipid emulsions) can be reduced by 97% simply by using IVT that does not contain DEHP.

Aside from bubble CPAP, respiratory DEHP exposures were mostly small, being near the baseline level observed in infants with no known exposures other than environmental. DEHP exposures from bubble CPAP appear to be drastically higher than any other tested respiratory devices other than ventilation through a DEHP-positive endotracheal tube [21]. Mask CPAP (without the bubbler) was not tested in this study, but we reported three patients in a prior study where urine metabolites in two infants receiving mask CPAP were below detection, whereas a bubble CPAP patient showed similar urine metabolite levels as seen in the present study [16]. These values are shown in the Appendix C.

Premature infants receive conventional IVF for treatment of hypoglycemia, hypovolemia, or as a carrier fluid for medication delivery. The DEHP exposure from commercial IVF appears relatively small, but can be eliminated by using DEHP-negative IVF and IVT. Recently, we reported a marked drop in neonatal hypertension in one center (which did not use bubble CPAP) when IVF in both antenatal and postnatal units inadvertently changed from a DEHP-positive fluid to a DEHP-negative IVF [28]. This observation suggests there may be benefit from reducing even small DEHP exposures.

HA fluid is usually delivered from containers not made of PVC. Components making up the HA fluid could come from a variety of containers and delivery devices. It is not clear what is responsible for the marked increase in DEHP delivery when HA fluid is administered through DEHP-positive tubing. The lipid emulsion containers we tested were made of glass, or plastic that did not contain DEHP. Our results were similar, but not as high as reported by Loff, who in 2004 showed the load of DEHP leached from lipid emulsions into infants could reach several milligrams per day when administered through IVT containing DEHP [29]. Loff advised that use of such tubing “should be abandoned for infusions in babies”. Our results show that advice has not yet been taken.

Aside from the DEHP exposures we evaluated with this study, other miscellaneous IV DEHP exposures are likely small. Saline flushes are often administered with prefilled syringes not constructed with PVC. We have not encountered IV cannula labeled DEHP-positive in the NICU, although they may exist. DEHP exposures from IV medications might be consequential if delivered from DEHP-positive IV bags, especially if large amounts of medications were delivered over long periods. Finally, blood products are usually delivered from DEHP-negative containers through microbore tubing (which is labelled DEHP-negative in our NICU). As with lipids, there are past reports that blood products administered through DEHP-positive IVT can deliver amounts of DEHP exceeding 150 mcg/kg/day [30]. More recently, progress is being made in eliminating DEHP from blood storage bags [31].

Phthalates other than DEHP are used in medical devices in other parts of the world as described by Wang in a recent review [1]. Alternatives to DEHP for use in medical devices have been rapidly evolving and include alternative phthalates, nonphthalate plasticizers, and alternative polymers [18,32]. Some alternative plasticizers such as di(isonyl)cyclohexane–1,2-dicarboxylate (DINCH) have eightfold less leaching into enteral solutions as compared to DEHP [18]. Still, when evaluating infusion devices labeled DEHP-free in Europe, Genay reported only two of nine tested medical devices were truly DEHP-free [33]. PVC-free polymers may prove safer alternative to PVC-based plastics.

Pediatric studies on the metabolic disposition of environmental chemical are exceedingly rare. Accidental exposure is often the only impetus for such assessment and while this sometimes occurs in the occupational environment, it has rarely occurred among infants or children. Given enzyme activity can be age-dependent, the use of adult data to calculate metabolic rates is a limiting factor. For example, in vitro human liver studies demonstrate strong evidence that CYP2C9 and CYP3A4 have a role in DEHP metabolism [34]. Importantly, expression of CYP2C9 and CYP3A4 does not reach adult levels until years after birth and not until puberty in some individuals [35]. Koch has suggested alterations in DEHP metabolism are largely due to the degradation rates of primary and secondary metabolites, but his method of integrating the major metabolites accounts for these differences [8]. To that point, our data did show distributions of metabolites with less MEHP and more MEOHP as compared to Koch’s adult data [8].

The strength of this study is that it provides new data that can be used in a targeted and practical way to reduce DEHP exposures and associated potential adverse effects in small infants. There were limitations of this study aside from the unknown variation of infant DEHP metabolism as compared to that in an adult. The indirect (in vivo) testing method was based on metabolite excretion in one single adult individual. Additionally, the duration of time to reach steady state excretion of a DEHP load for a premature infant is unknown and likely longer than that of an adult. Although not a study objective, we could not tell if certain low-level exposures were statistically different from one another given the small number of tests performed. Lastly, we cannot exclude there are other potential DEHP exposures in the NICU from devices unlabeled or wrongly labeled for DEHP content.

## 5. Conclusions

Although safe levels of DEHP exposure in premature infants have yet to be established, the concern for potential harm suggests hospitals pursue reduction of DEHP exposure when practical to do so. These results should be a guide to such an effort. In order of potential impact, avoiding bubble CPAP, and DEHP-positive IV tubing would have the greatest impact on reducing DEHP exposure. Most of these products are commercially available today. Lastly, adjustments to bubble CPAP systems to reduce DEHP exposure should be considered.

## Figures and Tables

**Table 1:** DEHP content (mcg/L) in three types of IV fluid, one type of HA fluid, and three types of lipid emulsion.

	Median	IQ Range	Range	Median	IQ Range	Range	Median	IQ Range	Range
Intravenous Product	From Container			DEHP-Negative IV Set			DEHP-Positive IV Set		
DEHP-negative IVF	0.0	0.0	0–0.2	0.0	0.0	0–0	5.2 *	12.1	2.4–15.0
DEH-unlabeled IVF	27.0	+	26–40	2.4	1.4	1.4–3.2	11.0 *	16.2	2.3–26.0
DEHP-positive IVF	560.0	+	560–620	32.0	24.1	7.6–40	15.0	24.8	4.4–38.0
HA fluid	5.1	+	3.4–13.0	6.7	13.1	0–16	500.0 *	1430	420–2300
Mixed lipid emulsion	1.9	4.4	0–5.9	0.0	0.0	0–0	9300 *	4300	6100–13,000
fish oil lipid emulsion	8.3	10.8	0–15	92.0	62.6	74–170	4000 *	3350	2100–6900
Soybean lipid emulsion	0.0	0.0	0.0	0.0	0.0	0.0	12,000 *	23,050	3400–45,000

+, unable to calculate interquartile range due to *n* = 3. *, Denoting signifant difference (*p*, 0.05) in median DEHP concentration between fluid without and with DEHP in the IV set using Wilcoxsen ranked sum test. IV, intravenous; IVF, intravenous fluid; DEHP, di-2-ethylhexyl phthalate; ND, not dectected; HA, hyperalimentation fluid, IQ, interquartile range.

**Table 2:** Daily DEHP estimated exposures of respiratory therapy device based on urine metabolites of DEHP.

Respiratory Device	*n*	Median (mcg/Day)	IQ Range (mcg/Day)
Bubble CPAP	5	7843.5 *	6500.5
Room Air (baseline)	5	25.5	42.5
HFNC	5	21.6	20.3
LFNC	1 +	7.3	NA
Ventilator with DEHP-negative ETT	5	61.4	174.1

+, Four samples excluded due to additional IVF received by the patient. *, signifant difference (*p* < 0.05) in median DEHP exposure between the baseline and other respiratory device using Wilcoxsen ranked sum test; CPAP, continuous positive airway pressure; HFNC, high-flow nasal cannula; IQ, interquartile range; LFNC, low-flow nasal cannula; DEHP, di-(2-ethylhexyl phthalate); ETT, endotracheal tube; NA, not able to calculate an interquartile range when N = 1.

**Table 3:** Demographics of 14 very low birthweight infants for whom daily and cumulative DEHP exposures were calculated.

Birthweight (Kg)	0.9
Postmenstrual age at birth (weeks)	27.7
Postmenstrual age at discharge from hospital (weeks)	43.0
Caucasian race (%)	100
Hispanic ethnicity (%)	43
Female gender (%)	29

**Table 4:** Mean cumulative DEHP exposures for 14 VLBW infants based on actual IV and respiratory exposures using above derived values for DEHP exposure for each device. All IV tubing was DEHP-positive in these patients.

Mean Cumulative DEHP Exposure by IV Product or Respiratory Device	Quantity (mL-Days)	Mass (mcg)	Totals (mcg/Kg)
Conventional intravenous fluid	454 mL	5	
Starter (initial) hyperalimentation	133 mL	67	
Hyperalimentation fluid	2283 mL	1141	
Lipid emulsions	274 mL	2847	
Total intravenous DEHP		4039	
Mechanical ventilator ^+^	23 days	6616	
Bubble CPAP	28 days	219,338	
NIPPV	2 days	127	
Low flow nasal cannula	1 day	4	
High flow nasal cannula	3 days	69	
Mean respiratory DEHP		221,369	
Mean IV + respiratory DEHP		230,207	
Mean IV + respiratory DEHP per Kg			182,369

^+^, using non-DEHP endotracheal tube; DEHP, Di-2-ethylhexyl phthalate; VLBW, Very low birth weight; IV, Intravenous; CPAP, Continuous positive airway pressure; NIPPV, Noninvasive positive pressure ventilation.

## Data Availability

Data in contained within the article and Appendix A, Appendix B and Appendix C.

## Appendix A. Intravenous Equipment Testing for DEHP in Commercial IV Fluid

**Material**	**Ref #**	**Lot #**	**Labelling re DEHP/PHT Yes/No/Not Labelled**	**Test Date**	**Volume** **mL**	**Result** **mcg/L**
DEHP-positive 0.9% NS # 1	same	04-005-JT	Yes	8/25/2019	1000	560
DEHP-positive 0.9% NS # 2	same	04-005-JT	Yes	8/25/2019	1000	560
DEHP-positive 0.9% NS # 3	same	04-005-JT	Yes	8/25/2019	1000	620
				Median		560
DEHP-unlabeled 0.9% NS #1	same	307082	Not labelled	9/5/2019	1000	26
DEHP-unlabeled 0.9% NS #2	same	307082	Not labelled	9/5/2019	1000	27
DEHP-unlabeled 0.9% NS #3	same	312058	Not labelled	9/5/2019	1000	40
				Median		27
DEHP-negative 0.9% NS # 2	same	J9J059	No	9/5/2019	1000	0.21
DEHP-negative 0.9% NS # 3	same	J9J059	No	9/5/2019	1000	0
DEHP-negative 0.9% NS # 4	same		No	5/28/2020	1000	0
				Median		0
DEHP-positive NS + SLB IV set #1	2426-0007	94-100-jt	Yes/No	10/5-7/19	1000	19
DEHP-positive NS + SLB IV set #2	2426-0007	94-100-jt	Yes/No	10/5-7/19	1000	28
DEHP-positive NS + SLB IV set #3	2426-0007	94-100-jt	Yes/No	10/5-7/19	1000	41
DEHP-positive NS + SLB IV set #4	2426-0007	01-022-JT	Yes/No	1/21-1/23-20	1000	7.6
DEHP-positive NS + SLB IV set #5	2426-0007	04-005-JT	Yes/No	1/22-1/24/20	1000	40
DEHP-positive NS + SLB IV set #6	2426-0007	04-005-JT	Yes/No	1/21-1/22/20	1000	36
	IV set #			Median		32.0
DEHP-negative NS + SL and OHSU micro	2426-0007	J9J059	All labelled NO	9/17-9/19/19	1000	0.13
through SL non-DEHP sets	2426-0007	J9J059	All labelled NO	9/17-9/19/19	1000	0
i.e both microbores	2426-0007	J9J059	All labelled NO	9/17-9/19/19	1000	0
				Median		0
DEHP-unlabeled NS + OHSU Ivset #1	2401-500	312058	Not labelled/Yes	9/17-9/19/19	1000	18
DEHP-unlabeled NS + OHSU Ivset #2	2401-500	312058	Not labelled/Yes	9/17-9/19/19	1000	20
DEHP-unlabeled NS + OHSU Ivset #3	2401-500	312058	Not labelled/Yes	9/17-9/19/19	1000	26
DEHP-unlabeled NS + OHSU Ivset #4	2401-500	312508	Not labelled/Yes	10/5-7/2019	1000	12
DEHP-unlabeled NS + OHSU Ivset #5	2401-500	312508	Not labelled/Yes	10/5-7/2019	1000	9.9
DEHP-unlabeled NS + OHSU Ivset #6	2401-500	312508	Not labelled/Yes	5/28-5/29/20	500	4.0
DEHP-unlabeled NS + OHSU Ivset #7	2401-500	312508	Not labelled/Yes	5/28-5/29/20	500	2.3
DEHP-unlabeled NS + OHSU Ivset #8	2401-500	312508	Not labelled/Yes	5/28-5/29/20	500	3.1
				Median		11.0
DEHP-negative NS + SLB IV set	2426-0007	J9J059	All labeled NO	9/5/2019	1000	0
DEHP-negative lot J9J059 for all	2426-0007	J9J059	All labeled NO	9/5/2019	1000	0
DEHP-negative NS + SLB IV set	2426-0007	J9J059	All labeled NO	9/5/2019	1000	0
				Median		0
DEHP-negative NS + OHSU Ivset	2401-500	J9J059	No/Yes (IV set)	9/17-9/19/19	1000	15
DEHP-negative NS + OHSU Ivset	2401-500	J9J059	No/Yes (IV set)	9/17-9/19/19	1000	15
DEHP-negative NS + OHSU Ivset	2401-500	J9J059	No/Yes (IV set)	9/17-9/19/19	1000	3.4
DEHP-negative NS + OHSU Ivset	2401-500	J8J119	No/Yes (IV set)	10/507/2019	1000	5.2
DEHP-negative NS + OHSU Ivset	2401-500	j8j119	No/Yes (IV set)	10/507/2019	1000	2.4
				Median		5.2
DEHP-unlabeled NS + SLB IV set #1	2426-0007	312058	Unlabelled/No	1/21-1/23-20	1000	2.9
DEHP-unlabeled NS + SLB IV set #2	2426-0007	312058	Unlabelled/No	1/21-1/23-20	1000	3.0
DEHP-unlabeled NS + SLB IV set #3	2426-0007	312058	Unlabelled/No	1/22-1/24-20	1000	2.6
DEHP-unlabeled NS + SLB IV set #4	2426-0007	312508	Unlabelled/No	1/21-1/22/20	1000	2.1
DEHP-unlabeled NS + SLB IV set #5	2426-0007	312058	Unlabelled/No	5/28-5/29/20	500	1.4
DEHP-unlabeled NS + SLB IV set #6	2426-0007	312058	Unlabelled/No	5/28-5/29/20	500	3.2
DEHP-unlabeled NS + SLB IV set #7	2426-0007	312058	Unlabelled/No	5/28-5/29/20	500	1.4
DEHP-unlabeled NS + SLB IV set #8 (#4)	2426-0007	312508	Unlabelled/No	5/28-5/29/20	500	2.1
				Median		2.4
DEHP-positive NS + OHSU IV set #1	2401-500	01-22-JT (1/2	Yes/YES	1/21-1/23/20	1000	15.0
DEHP-positive NS + OHSU IV set #2	2401-500	01-22-JT	Yes/YES	1/21-1/23/20	1000	31.0
DEHP-positive NS + OHSU IV set #3	2401-500	01-22-JT	Yes/YES	1/21-1/23/20	1000	38.0
DEHP-positive NS + OHSU IV set #4	2401-500	01-22-JT	Yes/YES	5/28-5/29/20	500	15.0
DEHP-positive NS + OHSU IV set #5	2401-500	01-22-JT	Yes/YES	5/28-5/29/20	500	4.4
				Median		15.0

## Appendix B

**DEHP Concentrations in Hyperalimentation Fluid a**			**Smof = Mixed Lipid Emulsion**							
**TPN and LIPID**	**DEHP Presence**		**Estimated**							
	**Yes/No**	**Yes/No**		**Duration**	**Volume**		**DEHP**		**LOD**	
	**In Bag**	**In**	**Start date**	**Hours**	**mL**		**mcg/L**		**mcg/L**	
HA neg #1	No	No	6/8/2020	24		300		0		4.2
HA neg#2	No	No	6/8/2020	24		450		4.4		0.3
HA neg # 3	No	No	6/8/2020	24		450		8.9		0.31
HA neg #4	No	No	6/16/2020	23.34		250		16		7.7
				Average			7.3			
				Median			6.7			
HA pos #1	No	Yes	6/9/2020	24 400			540			7
HA pos # 2	No	Yes	6/9/2020	24 400			420			3
HA pos #3	No	Yes	6/9/2020	24 400			460			3.1
HA pos # 4	No	Yes	6/17/2020	24 290			2300			31
				Average			930.0			
				Median			500.0			
HA plain 1	No	N/A	6/10/2020 N/A	400			5.1			0.31
HA plain 2	No	N/A	6/10/2020 N/A	450			3.4			0.26
HA plain 3	No	N/A	6/10/2020 N/A	650			13			0.17
				Average			7.166667			
				Median			5.1			
Omega neg1	No	No	6/8/2020	12 30			74			22
Omega neg2	No	No	6/8/2020	12 30			110			21
Omega neg3	No	No	6/8/2020	12 30			170			21
Omega neg4	No	No	6/8/2020	12 30			81			21
Omega neg5	No	No	6/8/2020	12 30			92			21
				Average			105.4			
				Median			92			
Omega pos1	No	Yes	6/8/2020	12 30			6900			73
Omega pos2	No	Yes	6/8/2020	12 40			2800			52
Omega pos3	No	Yes	6/8/2020	11.5 50			2100			25
Omega pos4	No	Yes	6/8/2020	11.34 40			4000			41
Omega pos5	No	Yes	6/8/2020	12 50			4700			38
				Average			4100			
				Median			4000			
Omega plain1	No	N/A	6/18/2020 NA	200			15			12
Omega plain2	No	N/A	6/18/2020 NA	200			8.3			6.1
Omega plain3	No	N/A	6/18/2020 NA	200			7.5			5.9
Omega plain4	No	N/A	6/18/2020 NA	200			14			12
Omega plain5	No	N/A	6/18/2020 NA	200			0			24
				Average			9.0			
				Median			8.3			
Smofneg 1	No	No	6/8/2020	24 60			0			37
Smofneg 2	No	No	6/8/2020	24 60			0			18
Smofneg 3	No	No	6/15/2020	18 40			0 verify			
Smofneg 4	No	No	6/16/2020	24 40			0			150
Smofneg 5	No	No	6/8/2020	24 60			0			37
				Ave			0			
				Median			0			
Smofpos 1	No	Yes	6/8/2020	24 40			9300			170
Smofpos 2	No	Yes	6/8/2020	24 50			11,000			160
Smofpos 3	No	Yes	6/17/2020	24 50			13,000			130
Smofpos 4	No	Yes	6/8/2020	24 55			9300			110
Smofpos 5	No	Yes	6/8/2020	24 50			6100			130
				Ave			9740			
				Median			9300			
Smof plain 1	No	N/A	6/10/2020 NA	90			5.9			5.1
Smof plain 2	No	N/A	6/10/2020 NA	90			1.9			1.2
Smof plain 3	No	N/A	6/10/2020 NA	95			0			47
Smof plain 4	No	N/A	6/10/2020 NA	95			0			5.7
Smof plain 5	No	N/A	6/10/2020 NA	95			2.9			2.3
				Ave			2.1			
				Median			1.9			
Soybean neg 1	No	No	6/16/2020	23.17 40			0			50
Soybean neg 2	No	No	6/16/2020	23.08 45			0			39
Soybean neg 3	No	No	6/16/2020	23.17 40			0			36
Soybean neg 4	No	No	6/16/2020	23.58 35			0			30
Soybean neg 5	No	No	6/16/2020	23.67 30			0			150
				Ave			0			
				Median			0			
Soybean pos 1	No	Yes	6/17/2020	24 70			3400			48
Soybean pos 2	No	Yes	6/17/2020	24 80			9500			160
Soybean pos 3	No	Yes	6/17/2020	24 50			45,000			370
Soybean pos 4	No	Yes	6/17/2020	24 80			12,000			110
Soybean pos 5	No	Yes	6/17/2020	24 40			14,000			200
				Ave			16,780			
				Median			12,000			
Soybean plain 1	No	N/A	6/18/2020 NA	95			0			92
Soybean plain 2	No	N/A	6/18/2020 NA	95			0			46
Soybean plain 3	No	N/A	6/18/2020 NA	95			0			45
Soybean plain 4	No	N/A	6/18/2020 NA	300			0			28
				Ave			0			
				Median			0			

## Appendix C. Urine Metabolites for 21 Urine Samples with Estimates of Daily DEHP Intake

	**Days on Therapy**		**Day of Life (Days)**	**Post Conception al Age (Days)**	**Weight at Sampling (kg)**				**Urine Creatinine (mg/dL)**	**24 h Urine Creatinine Based on Age (mg/kg/Day)**	**Calculated DEHP Based on Age (mcg/Day)**	**DEHP Minus Median Baseline (mag/Day)**	**DEHP Minus Median in Last 24 h (mL)**			**DEHP from Convention IV Fluid Last 24 h (mcg)**
**Subject ID**	**Time**	**Modali**				**MEHP (ng/mL)**	**MEOHP (ng/mL)**	**MEHHP (ng/mL)**								
ID 1 30-AUG-19	15:00	27 CPAP	27	228	1.885	494	7550	8000	8.52	9.60	10,280.1	10,254.6	0			0
ID 1 03-SEP-19	17:30	31 CPAP	31	231	2.035	439	4490	4580	7.16	9.64	7869.0	7843.5	0			0
ID 1 04-SEP-19	12:00	32 CPAP	32	232	2.115	321	5870	6290	9.45	9.66	8137.4	8111.9	0			0
ID 3 19-SEP-19	16:00	15 CPAP	16	212	1.03	1050	10,100	14,000	20.2	9.36	3626.6	3601.1	0			0
ID 4 20-SEP-19	23:00	3 CPAP	3	230	1.87	1290	1840	6130	28.3	9.63	1790.5	1765.0	5.4			0.1
									CPAP	Median	7869.0	7843.5				
ID 1 17-SEP-19	20:15	8 RA	45	246	2.75	11.8	89.5	107	7.18	9.86	237.8		0			0
ID 2 03-SEP-19	14:39	5 RA	59	270	2.6	2.21	11.4	14.9	7.3	10.21	31.4		0			0
ID 3 10-OCT-19	3:00	8 RA	37	233	1.62	9.08	111	81.9	14.6	9.67	65.4		0			0
ID 3 23-OCT-19	3:00	21 RA	50	246	2.045	1.23	11.2	8.08	6.37	9.86	19.6		0			0
ID 5 08-OCT-19	23:00	17 RA	20	247	2.665	2.06	6.9	3.09	7.61	9.88	12.7		0			0
									Baseline IQ range	Median	25.5 42.5					
ID 6 13-NOV-19	13:00	16 HFNC	26	241	1.87	3.13	18.4	14.0	5.51	9.79	35.7			10.2	0	0
ID 6 14-NOV-19	17:00	17 HFNC	27	242	1.87	4.02	42.3	31.4	14.7	9.80	29.3			3.8	0	0
ID 7 13-NOV-19	17:00	5 HFNC	26	243	1.875	2.43	29.9	26.7	6.97	9.82	47.1			21.6	0	0
ID 7 18-NOV-19	14:30	10 HFNC	31	248	2.115	2.09	25.3	22.3	5.82	9.89	53.9			28.4	0	0
ID 7 22-NOV-19	2:30	14 HFNC	35	251	2.35	2.07	24.4	23.1	6.77	9.94	51.6			26.1	0	0
									HFNC	Median	47.1		21.6			
										IQ range	see T28		20.3			
ID 12 30-DEC-2019	14:08	6 Vent	55	236	1.25	1.88	21.2	25.7	2.8	9.71	63.9		38.4		0	0
ID 12 04-MAR-2020	20:00	11 Vent	119	302	2.69	12.6	52.3	49.8	7.79	10.69	128.2		102.7		0	0
ID 12 06-MAR-2020	5:00	13 Vent	121	304	2.85	7.53	33.3	24.8	7.35	10.71	82.6		57.1		0	0
ID 12 26-NOV-2019	4:00	4 Vent	21	203	0.665	34.5	454	740	6.21	9.23	366.6		341.1		56.3	0.6
ID 12 27-NOV-2019	9:15	5 Vent	22	204	0.735	4.97	95.7	176	6.53	9.24	86.9		61.4		70.0	0.8
Vent										Median	86.9		61.4			
**IQ Range**																
ID 2 29-AUG-2019	17:00	14 Nasal 13.42857 mean 13 median 3 min	54	275	2.445	4.13	19.1	21.1 LFNC	10.3	10.29	32.8		7.3		0	0
2014 study [10]		NIPPV		198	0.915	0	18	49	7.5	9.16	22.6				24	0.8
		HFNC		212	1.46	0	25	45	8.8	9.36	32.8				49	1.6
		HFNC		210	0.663	<LOD	ssay faile	ssay		9.33	assay failed				0	0.0
		Vent		193	0.784	32	69	0	6.1	9.08	36.0				10	0.3
		mask cpap		212	1.19	<LOD	<LOD	<LOD		9.36	<LOD				0	0.0
		mask cpap		188	0.884	<LOD	<LOD	<LOD		9.01	<LOD				12	0.4
		bubble cpap		222	1.44	650	1600	0	7.5	9.51	2651.3				0	0.0
		Room air		212	1.46	<LOD	<LOD	<LOD		9.36	<LOD				0	0.0
